# Supplementary material for: Transcriptional dynamics uncover the role of BNIP3 in mitophagy during muscle remodeling in Drosophila
Source: eLife. 2025 Aug 13;14:RP105834. doi: 10.7554/eLife.105834 (PMC12349898; doi:10.7554/eLife.105834)
Supplement: Figure 5—source data 1. [file elife-105834-fig5-data1.zip › Figure 5-souce data1/Figure 5C_Source data.pdf]

3xHA-mCherry-Atg18a (input 1%)

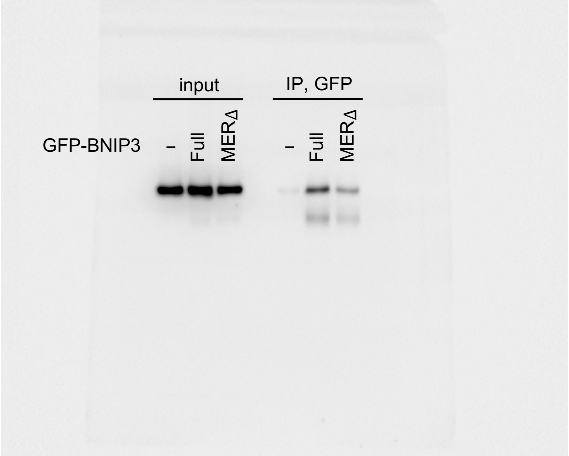

GFP-BNIP3 (input 10%)

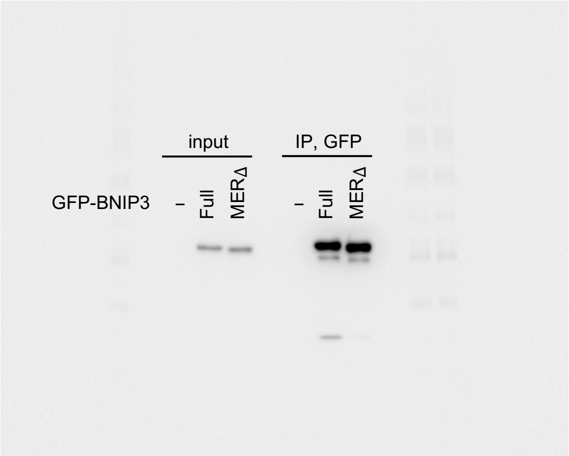

Composite data

3xHA-mCherry-Atg18a (input 1%)

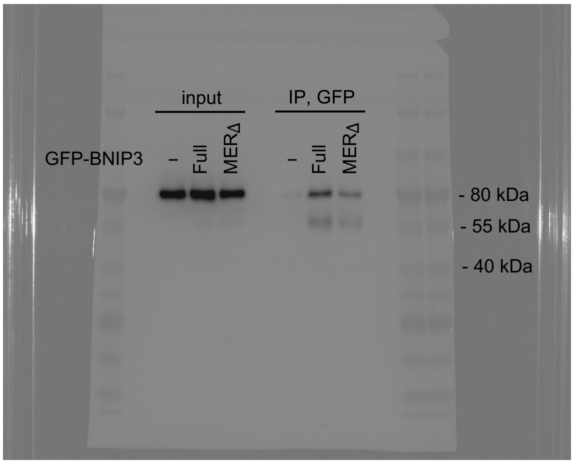

GFP-BNIP3 (input 10%)

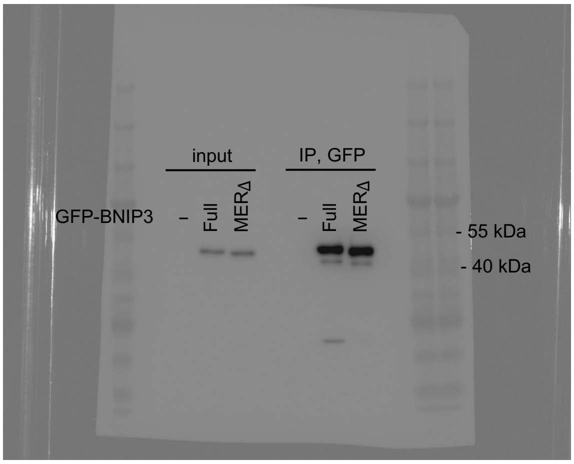

Figure 5C, Source Data 1. The top panels display the original membrane images corresponding to Figure 5C. The bottom panels show the same membranes overlaid with molecular weight markers. The three rightmost lanes represent input samples, while the three leftmost lanes show samples immunoprecipitated with anti-GFP antibodies.
