# Supplementary material for: Transcriptional dynamics uncover the role of BNIP3 in mitophagy during muscle remodeling in Drosophila
Source: eLife. 2025 Aug 13;14:RP105834. doi: 10.7554/eLife.105834 (PMC12349898; doi:10.7554/eLife.105834)
Supplement: Figure 5—source data 1. [file elife-105834-fig5-data1.zip › Figure 5-souce data1/Figure 5E_Source data.pdf]

IB:GFP

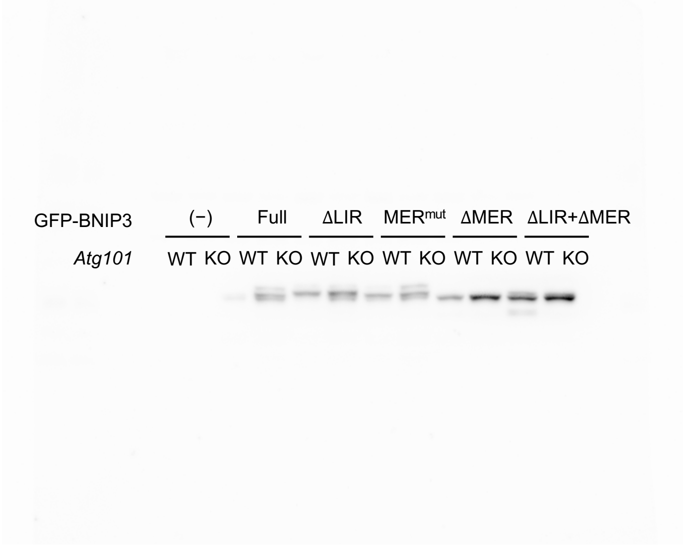

Composite data
IB:GFP

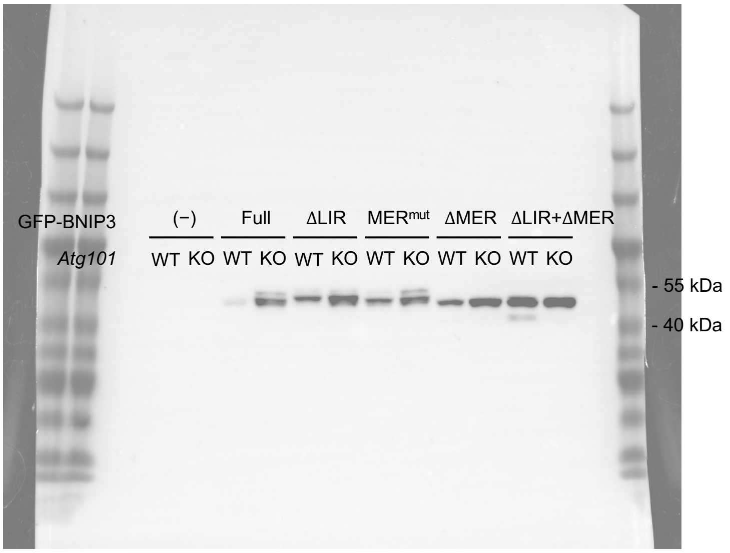

CBB stain

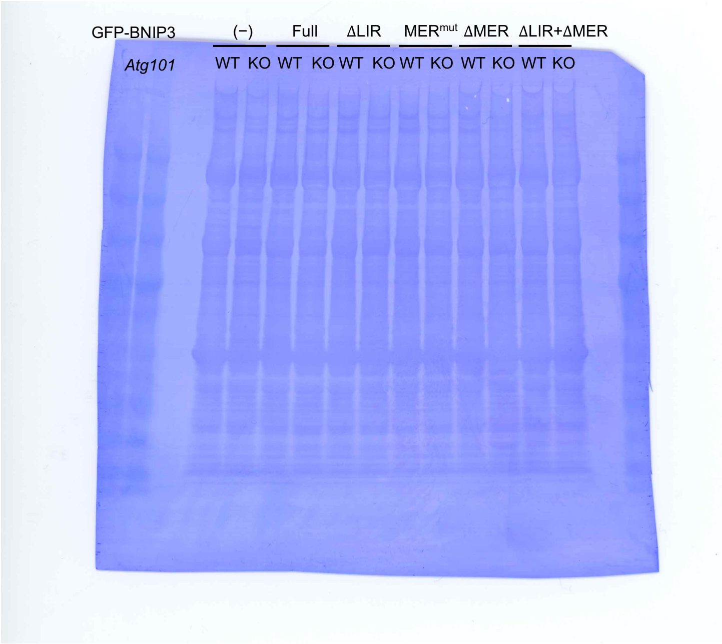

Figure 5E, Source Data 1. The top image shows the original membrane corresponding to Figure 5E. The middle image displays the same membrane overlaid with molecular weight markers. The bottom image shows the membrane after CBB staining. Lane labels are indicated in each image.
