# Supplementary figures and images for: Transcriptional dynamics uncover the role of BNIP3 in mitophagy during muscle remodeling in Drosophila
Source: eLife. 2025 Aug 13;14:RP105834. doi: 10.7554/eLife.105834 (PMC12349898; doi:10.7554/eLife.105834)

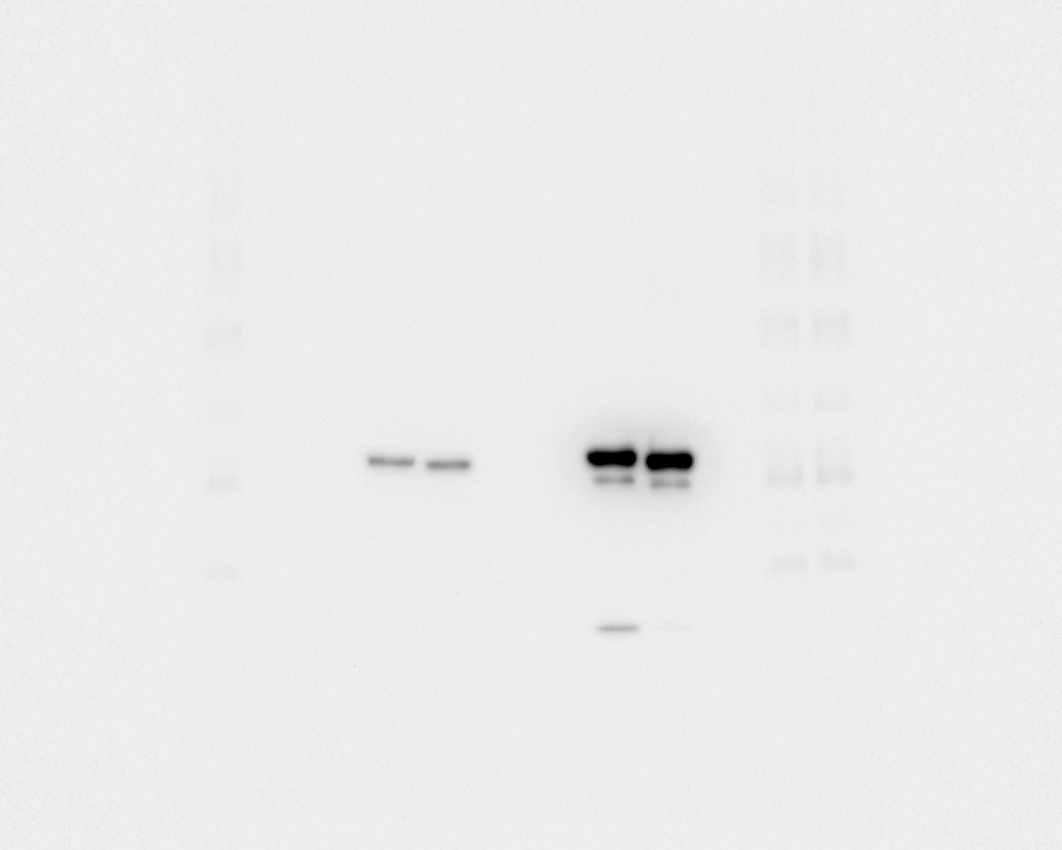

Supplement: Figure 5—source data 2. [file elife-105834-fig5-data2.zip › Figure 5-souce data2/Figure 5C_GFP-BNIP3 (Chemiluminescence).tif]

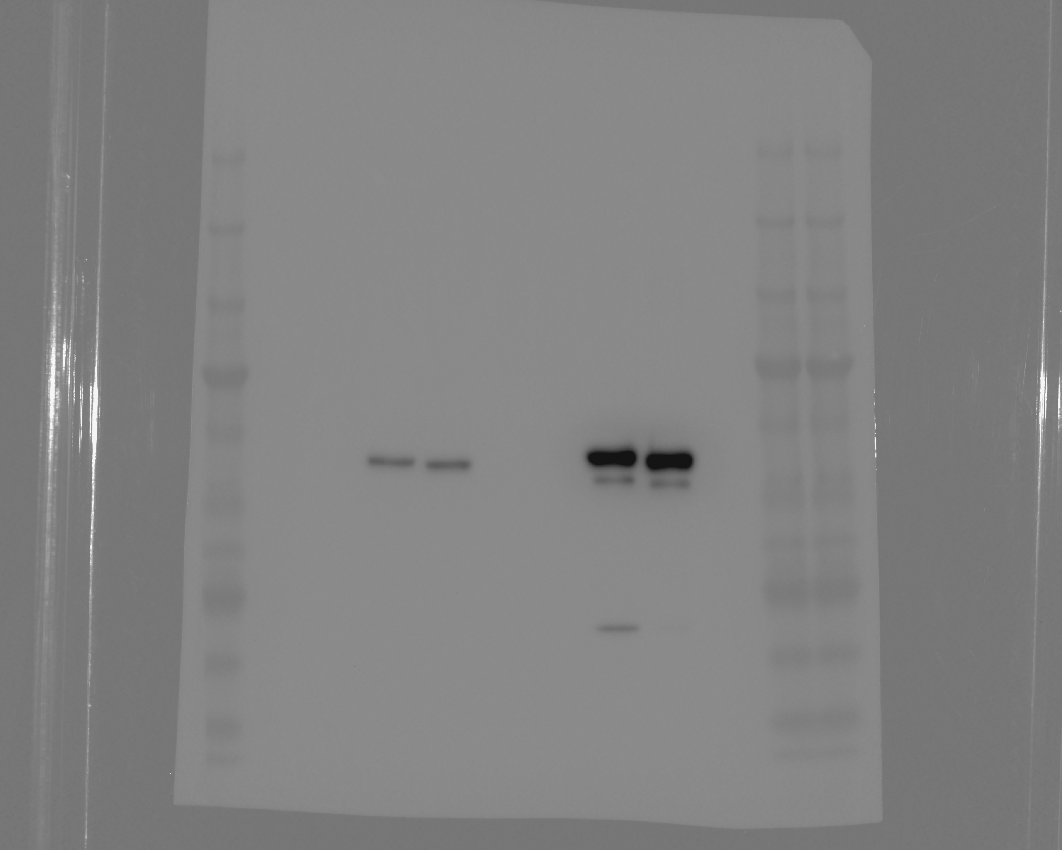

Supplement: Figure 5—source data 2. [file elife-105834-fig5-data2.zip › Figure 5-souce data2/Figure 5C_GFP-BNIP3(Composite).tif]

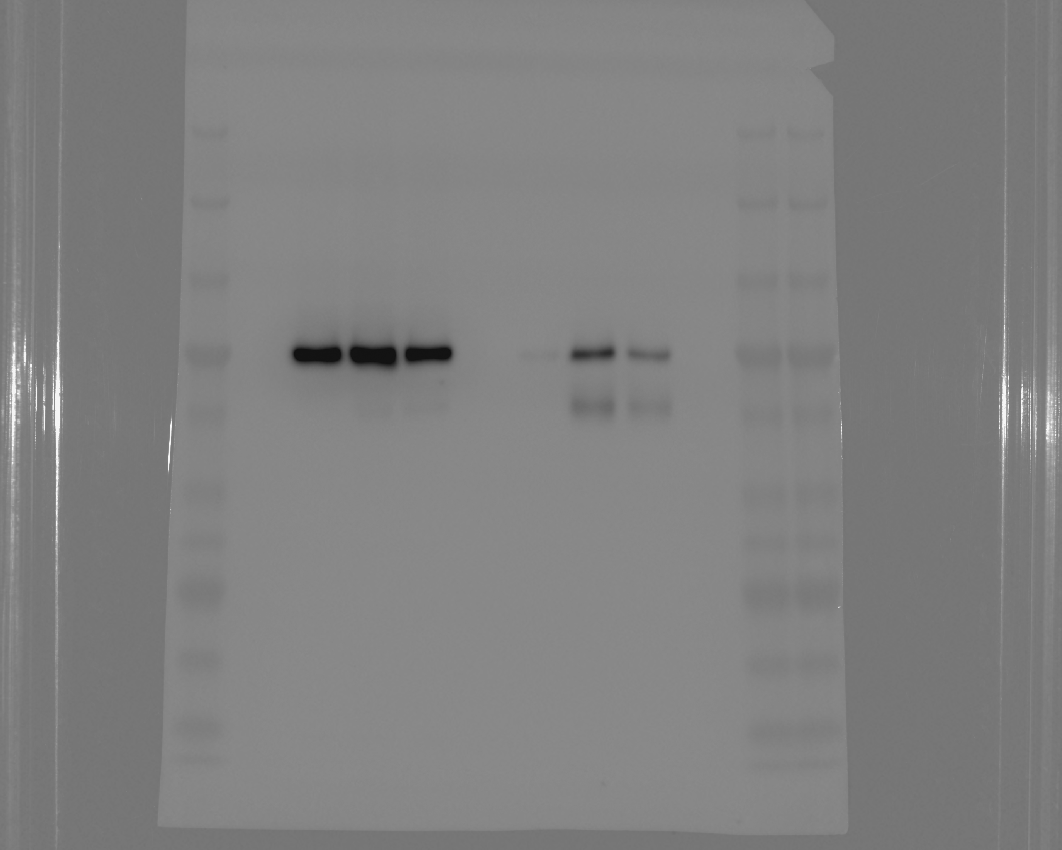

Supplement: Figure 5—source data 2. [file elife-105834-fig5-data2.zip › Figure 5-souce data2/Figure 5C_3HA-mCherry-Atg18 (Composite).tif]

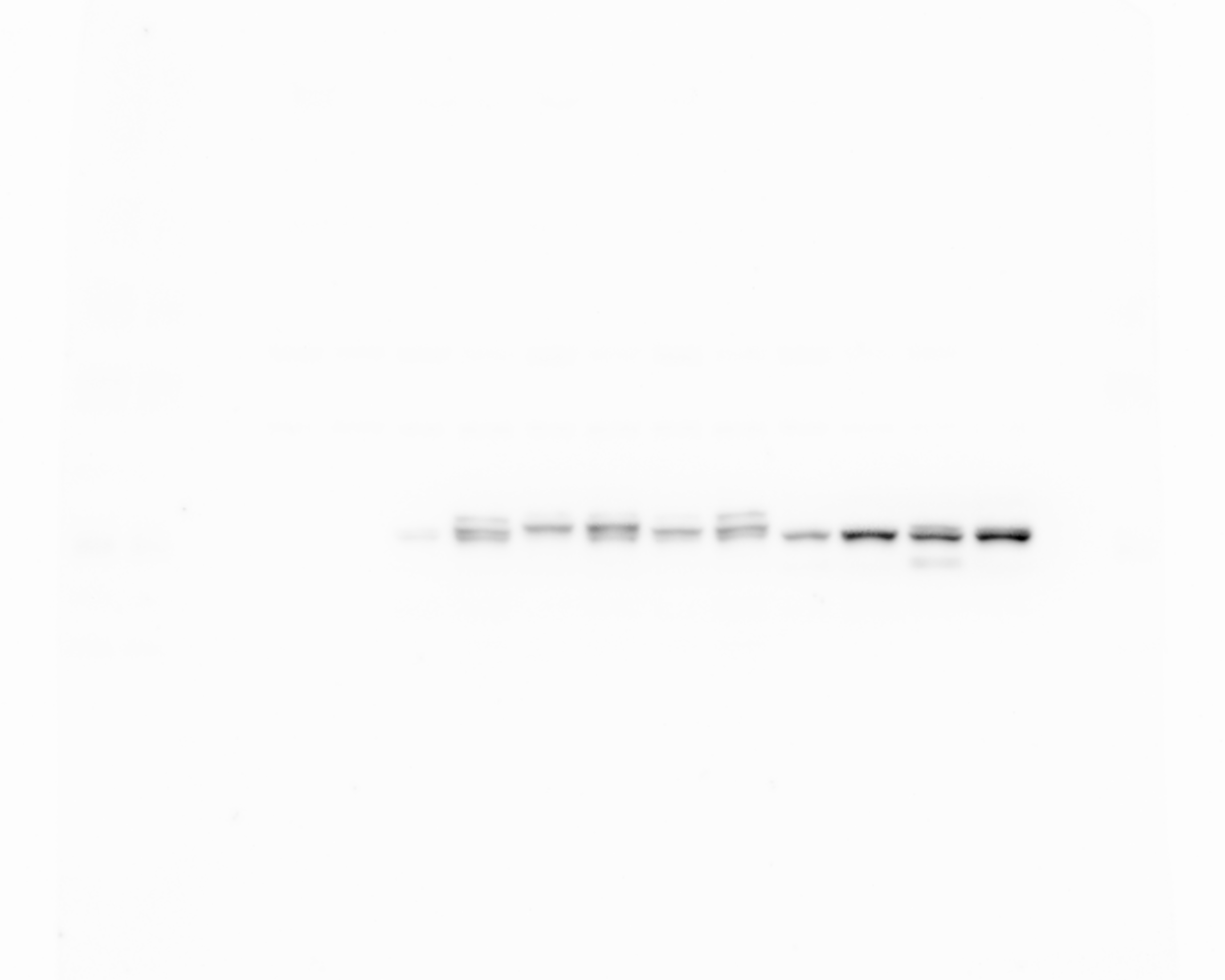

Supplement: Figure 5—source data 2. [file elife-105834-fig5-data2.zip › Figure 5-souce data2/Figure 5E_GFP-BNIP3 (Chemiluminescence).raw16.tif]

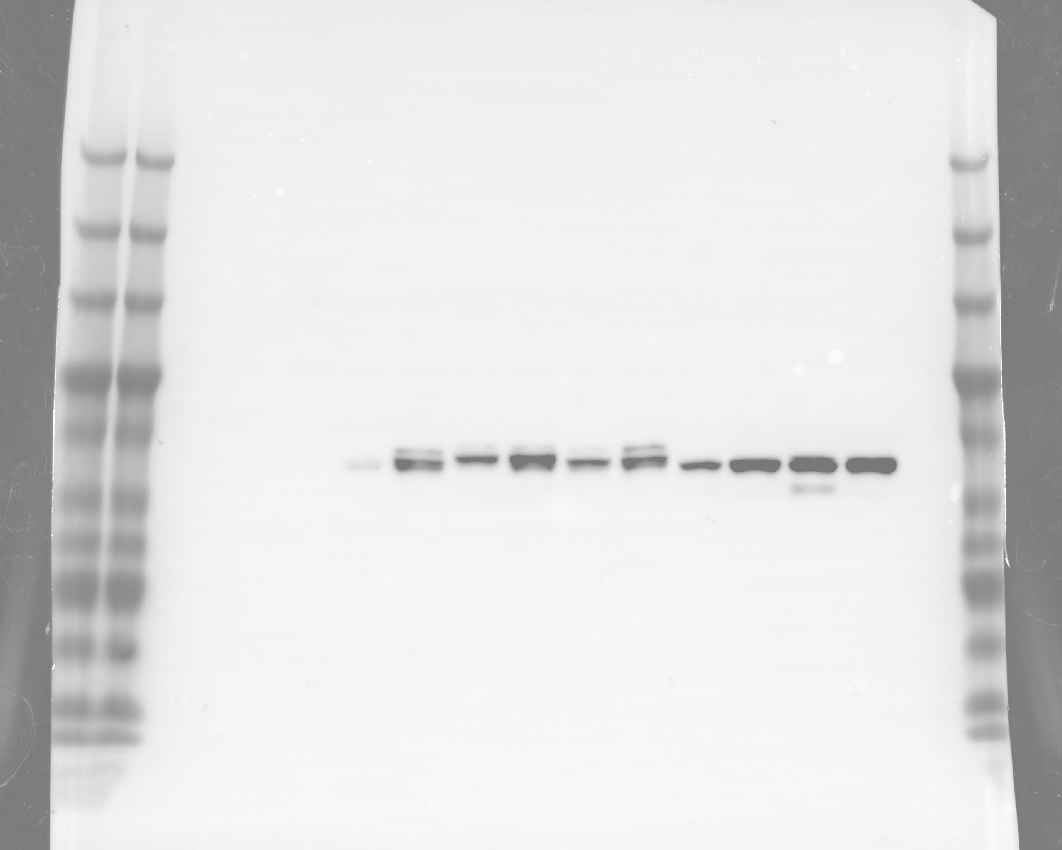

Supplement: Figure 5—source data 2. [file elife-105834-fig5-data2.zip › Figure 5-souce data2/Figure 5E_GFP-BNIP3 (Composite).tif]

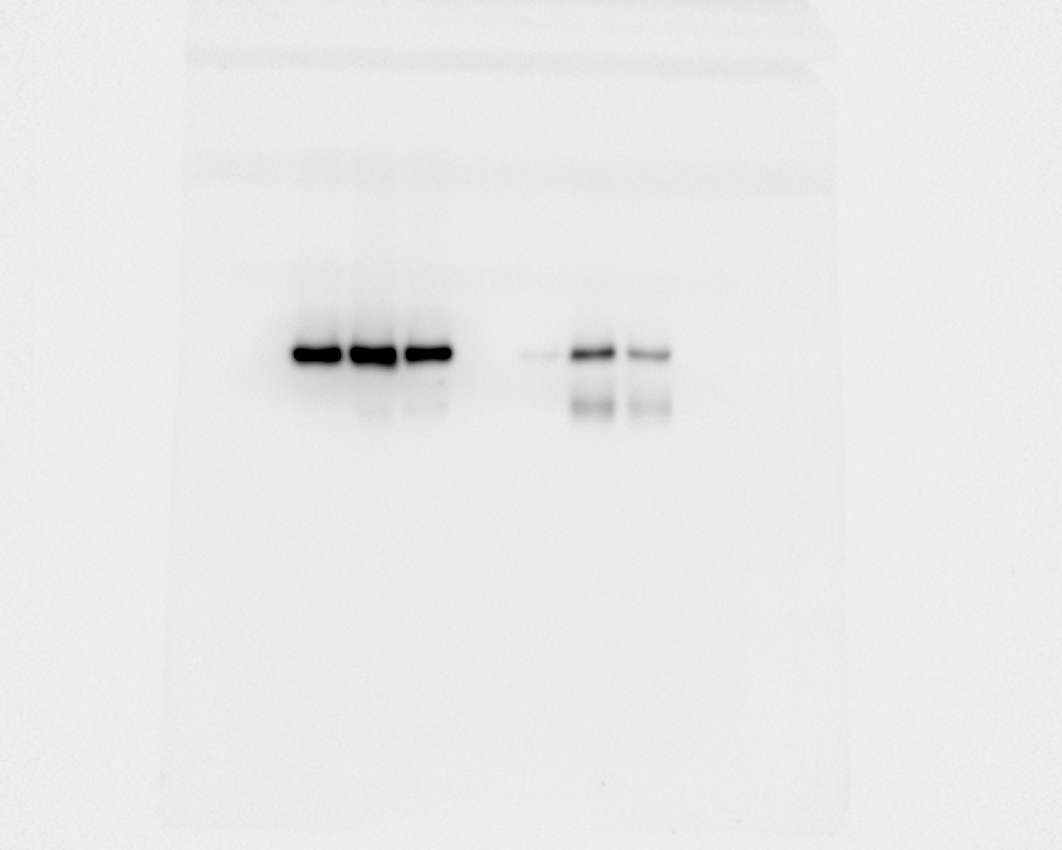

Supplement: Figure 5—source data 2. [file elife-105834-fig5-data2.zip › Figure 5-souce data2/Figure 5C_3HA-mCherry-Atg18 (Chemiluminescence).tif]

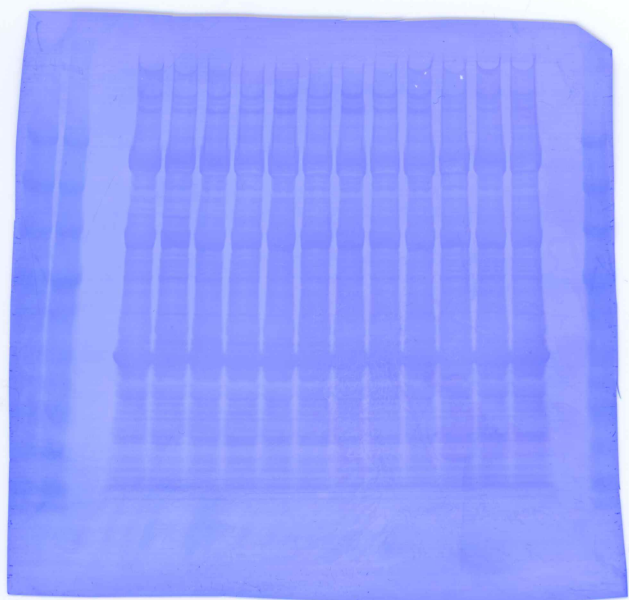

Supplement: Figure 5—source data 2. [file elife-105834-fig5-data2.zip › Figure 5-souce data2/Figure 5E_CBB.pdf]
